# CDK1 may promote breast cancer progression through AKT activation and immune modulation

**DOI:** 10.3389/fonc.2025.1591706

**Published:** 2025-11-06

**Authors:** Huanhong Zeng, Minxue Zhuang, Bochuan Liang, Feili Cai, Mengbo Lin, Ruijuan Wang, Ruo Wang, Hui Zhang

**Affiliations:** 1 Shengli Clinical Medical College of Fujian Medical University, Department of Breast Surgery, Fujian Provincial Hospital, Fuzhou University Affiliated Provincial Hospital, Fuzhou University, Fuzhou, China; 2 Nanchang Medical College, Nanchang, China

**Keywords:** Cdk1, breast cancer, AKT signaling, immune infiltration, prognosis, biomarker

## Abstract

**Background:**

Cyclin-dependent kinase 1 (CDK1) plays a crucial role in regulating the cell cycle, yet its clinical relevance and molecular mechanisms in breast cancer remain insufficiently characterized. This study aimed to comprehensively evaluate CDK1 expression, prognostic value, and biological functions in breast cancer through integrated bioinformatics and experimental analyses.

**Methods:**

Transcriptomic and clinical data from The Cancer Genome Atlas (TCGA) were analyzed to assess CDK1 expression, diagnostic efficacy, and survival associations. Immune infiltration and tumor mutation burden (TMB) were evaluated using TIMER and CIBERSORT algorithms. Single-cell RNA sequencing data from TISCH2 were employed to examine cell-type-specific expression. Functional experiments, including shRNA-mediated CDK1 knockdown, Western blotting, and CCK-8 assays, were performed to validate its biological role in MDA-MB-231 cells.

**Results:**

CDK1 expression was elevated in breast cancer tissues compared with normal controls and exhibited high diagnostic accuracy (AUC = 0.978). Elevated CDK1 levels were associated with HER2-, ER-, and PR-negative subtypes and enriched in Basal-like breast cancer. Patients with high CDK1 expression showed poorer disease-specific survival (HR = 1.67, p = 0.024). Immune analysis revealed positive correlations between CDK1 and immune cell infiltration, particularly CD4+ memory T cells, CD8+ T cells, etc. as well as a moderate association with TMB. Single-cell analysis indicated that CDK1 was preferentially expressed in CD8+ T cells and M1 macrophages. Mechanistically, CDK1 knockdown reduced AKT phosphorylation and downregulated Cyclin D1, A, and E1, leading to suppressed proliferation of breast cancer cells.

**Conclusion:**

CDK1 acts as a multifaceted oncogenic factor in breast cancer, contributing to tumor growth and immune modulation. Its overexpression is linked to poor prognosis and enhanced immune infiltration, underscoring its potential as a diagnostic and therapeutic target. Targeting CDK1 or its downstream signaling pathways may offer novel strategies, particularly for aggressive subtypes such as Basal-like or triple-negative breast cancer.

## Introduction

Cell cycle regulation is a key biological process that controls cell division, growth, and replication. CDK1, a cyclin-dependent kinase, plays a crucial role in driving the G2/M transition of the cell cycle by forming complexes with cyclins, primarily cyclin B1, to initiate mitosis. Dysregulation of CDK1 is frequently linked to uncontrolled cell proliferation and tumor development, positioning it as a vital player in cancer biology (1, 2). In particular, breast cancer, one of the most prevalent cancers in women, exhibits abnormal regulation of many cell cycle regulators (3–5). In fact, recent advances in genomics have facilitated the identification of important molecular markers implicated in breast cancer progression, among which CDK1 has emerged as a rising star (6–8). Beyond its canonical role in cell cycle progression, CDK1 may also participate in other crucial pathways, including DNA damage repair and modulation of the tumor immune microenvironment (9, 10). These findings suggest that CDK1 may play a multifaceted role in tumor progression, and its inhibition could offer multiple therapeutic benefits (11, 12). Breast cancer has been considered as one of the most commonly diagnosed cancer types in female globally (13–15). Similar to many of the other cancer types, breast cancer also has a high heterogeneity. Thereby, it is subdivided into many molecular subtypes, each with distinct genetic features, clinical outcomes, and treatment responses (16–21). Such molecular classifications are primarily based on the expression of hormone receptors, including estrogen receptor (ER), progesterone receptor (PR), and human epidermal growth factor receptor 2 (HER2). Furthermore, a set of 50 biomarkers in termed of PAM50 can categorize breast cancer into luminal A, luminal B, HER2-positive, basal, and normal-like subtypes (22). With such a complexity of breast cancer, although the mortality rate has decreased in recent years, the treatment modality of breast cancer remains a huge challenge and is subject to uncertainty. Therefore, the discovery of new molecular mechanisms is crucial for the development of therapeutic targets. Therefore, in the present study, we analyzed CDK1 expression in breast cancer using RNA-sequencing data from The Cancer Genome Atlas (TCGA) to compare its expression in normal and tumor tissues. We also evaluated the diagnostic potential of CDK1 by constructing receiver operating characteristic (ROC) curves. In parallel, we assessed the functional role of CDK1 in breast cancer cell viability through CDK1 knockdown experiments. Additionally, we explored the relationship between CDK1 expression and different breast cancer subtypes, focusing on ER, PR, HER2 status, and PAM50 molecular classifications.

## Materials and methods

### Data collection and processing

**Figure 1:**
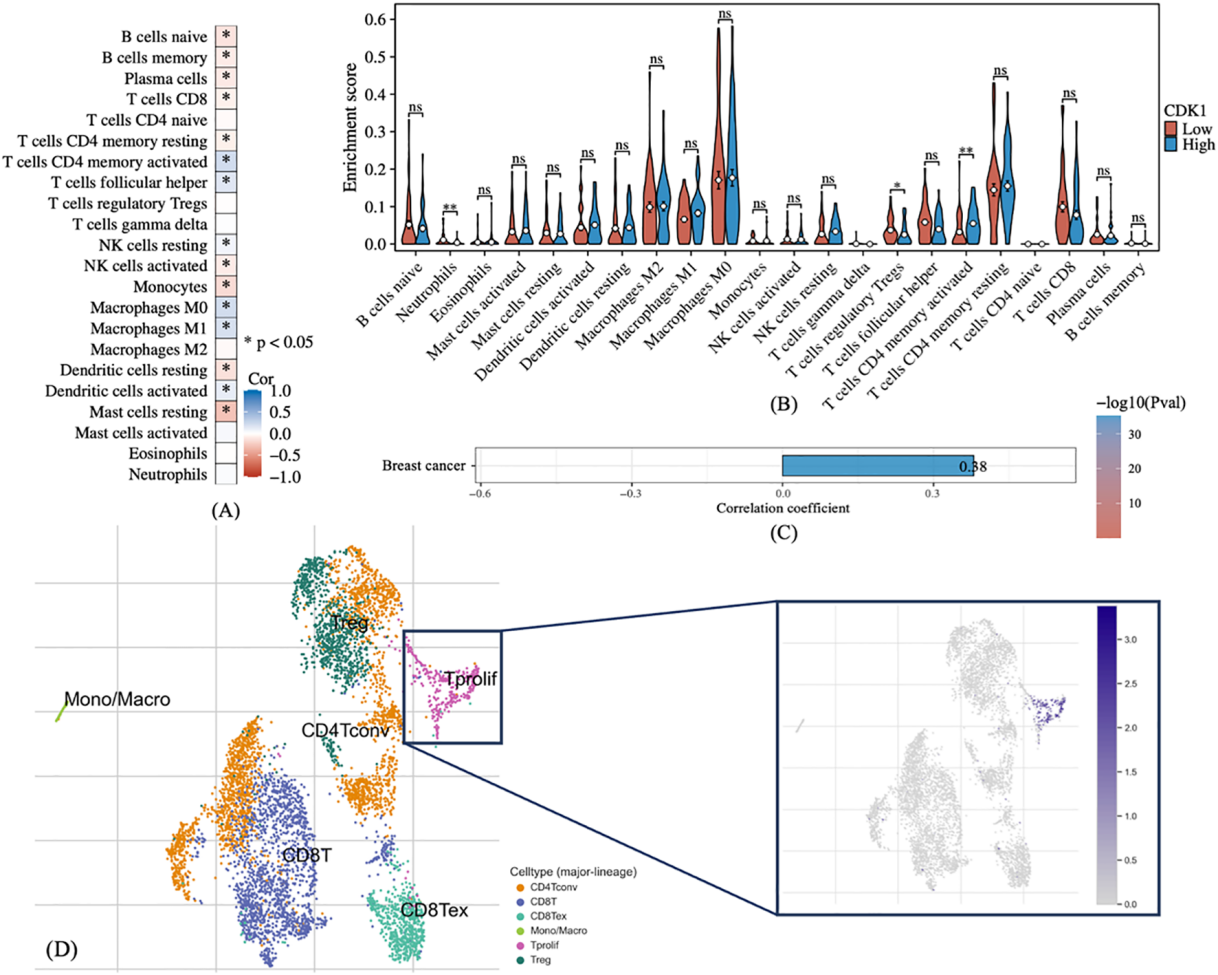
Interaction between CDK1 and the tumor immune microenvironment of breast cancer. **(A)** Correlation heatmap between CDK1 expression and various immune cell types in the tumor microenvironment. **(B)** Violin plot demonstrating the difference of immune cell infiltration between high- and low- CDK1 expression groups. **(C)** Correlation between CDK1 expression and TMB, indicating a positive relationship. **(D)** Single-cell RNA-seq analysis showing a strong association between CDK1 expression and proliferating T cells. Spearman correlation was used to evaluate associations between CDK1 expression and immune parameters. The Wilcoxon rank-sum test was applied for group comparisons. *: P-values < 0.05, **: P-values < 0.01. ns, Not significant.

Bulk RNA-sequencing data for Breast invasive carcinoma (TCGA-BRCA) were obtained from the TCGA database (release 2022.08). A total of 1,226 RNA-seq samples processed using the STAR workflow and normalized to TPM values were included. Among them, 113 were adjacent normal samples, and 1,226 were tumor samples. Clinical data were available for 1,098 cases, while 1,198 RNA-seq profiles contained corresponding or partially matched clinical information. Additionally, 18 RNA-seq samples originated from the same patient and were used only for consistency checks to avoid duplication bias. For survival and immune correlation analyses, only samples with both RNA-seq and complete clinical information (n = 1,098) were retained. The single-cell RNA-seq data presented in **Figure 1D** were obtained from the TISCH2 database (http://tisch.comp-genomics.org/), which includes annotated immune and stromal cell populations from human breast tumors. CDK1 expression levels were visualized across CD8+ T cells, M1 macrophages, and regulatory T cells using normalized log2(TPM + 1) values provided by the platform. Detailed source data could be found on its sub-webpages entitled “Documentation” (http://tisch.comp-genomics.org/documentation/). All statistical analyses were performed using R software (version 4.2.1). The following packages were employed: ggplot2 (v3.4.4) for data visualization, stats (v4.2.1) and car (v3.1-0) for statistical tests. Appropriate statistical methods were selected according to the distribution and characteristics of the data; analyses not meeting statistical assumptions were excluded. The Kruskal–Wallis test was used to compare differences between multiple groups. If not specified, in all the mentioned analyses, P-values < 0.05 considered statistically significant and represented by * in the figures. Furthermore, **: P-values < 0.01, ***: P-values < 0.001, if any.

### Differential expression analysis

The differentially expressed genes were extracted using the Limma algorithm, and the filtering conditions were set to Adjusted P-values < 0.05, |Log2 Fold Change| > 1.

### Survival analysis

The mean CDK1 expression level was used to divide the samples into high expression group and low expression group, and Kaplan-Meier analysis was performed using the R package “survival”.

### Tumor immune microenvironment assessment

The Tumor Immune Assessment Resource (TIMER) and CIBERSORT algorithms were used to compare the infiltration levels of various immune cells between the CDK1 high expression and low expression groups.

### Cell culture

MCF-10A cells were maintained in DMEM/F12 medium (Gibco) supplemented with 5% horse serum, 20 ng/mL epidermal growth factor (EGF), 0.5 μg/mL hydrocortisone, 100 ng/mL cholera toxin, and 10 μg/mL insulin. MDA-MB-231 cells were cultured in high-glucose DMEM (Gibco) containing 10% fetal bovine serum (FBS) and 1% penicillin-streptomycin. All cells were incubated at 37 °C in a humidified atmosphere containing 5% CO_2_. Cells were routinely subcultured at 70–80% confluence using 0.25% trypsin-EDTA, and all experiments were performed with cells passaged for fewer than six months. Cell line authentication was confirmed by short tandem repeat (STR) profiling, and mycoplasma contamination was excluded by PCR-based testing prior to experimental use.

### Plasmid transfection

To knock down CDK1 expression in MDA-MB-231 cells, a CDK1 shRNA expression plasmid (200 ng/μL) was transfected using Lipo8000™ transfection reagent (Beyotime, China) according to the manufacturer’s instructions. Briefly, MDA-MB-231 cells were seeded into 6-well plates at a density of 2 × 10^5^ to 7 × 10^5^ cells per well and cultured for 18–24 hours to reach 70–80% confluence at the time of transfection. On the day of transfection, the culture medium was replaced with 2 mL of fresh complete medium (high-glucose DMEM supplemented with 10% fetal bovine serum and 1% penicillin-streptomycin). For each well, 125 μL of serum- and antibiotic-free high-glucose DMEM was added to a sterile microcentrifuge tube, followed by 100 pmol of CDK1 shRNA plasmid (equivalent to 500 ng in 2.5 μL). The mixture was gently pipetted to mix. Then, 4 μL of Lipo8000™ was added, and the mixture was gently pipetted again. The transfection complex was incubated at room temperature for 20 minutes to allow complex formation. The 125 μL transfection mixture was then added dropwise to each well and evenly distributed by gently rocking the plate. Cells were incubated under standard conditions (37 °C, 5% CO_2_) for continued culture. At 48 hours post-transfection, GFP fluorescence was observed using a fluorescence microscope to evaluate transfection efficiency.

### Real-time quantitative PCR

Total RNA was extracted using Trizol reagent (Vazyme), and its concentration and purity were determined with a spectrophotometer. Complementary DNA (cDNA) synthesis was performed using the PrimeScript RT reagent kit (Vazyme) following the manufacturer’s protocol. Quantitative PCR was conducted on a LightCycler 96 Real-Time PCR System (Roche). Gene expression levels were calculated using the 2^−ΔΔCt method, with GAPDH serving as the internal control. All reactions were carried out in triplicate to ensure reproducibility. The primer sequences for CDK1 were as follows: Forward: 5’-CCTTTAGCGCGGATCTACC-3’, Reverse: 5’-GGAACCCCTTCCTCTTCACT-3’.

### Cell viability assay

Cell viability was assessed using the CCK-8 Cell Counting Kit (Vazyme). Cells were seeded in a 96-well opaque plate, and luminescence was measured using the GloMax-Multi Detection System (Promega) according to the manufacturer’s instructions. The percentage of cell viability was calculated based on the recorded luminescence.

### Statistical analysis

All experiments were conducted at least three times, and data are presented as mean ± standard error of the mean (SEM). Statistical analysis was performed using GraphPad software. Student’s t-test was used for comparisons between groups, with P-values < 0.05 considered statistically significant. Wilcoxon rank-sum tests were employed to assess differences in continuous target or class variables in cell subgroups.

## Results

### CDK1 is abnormally upregulated in breast cancer tumor tissues

**Figure 2:**
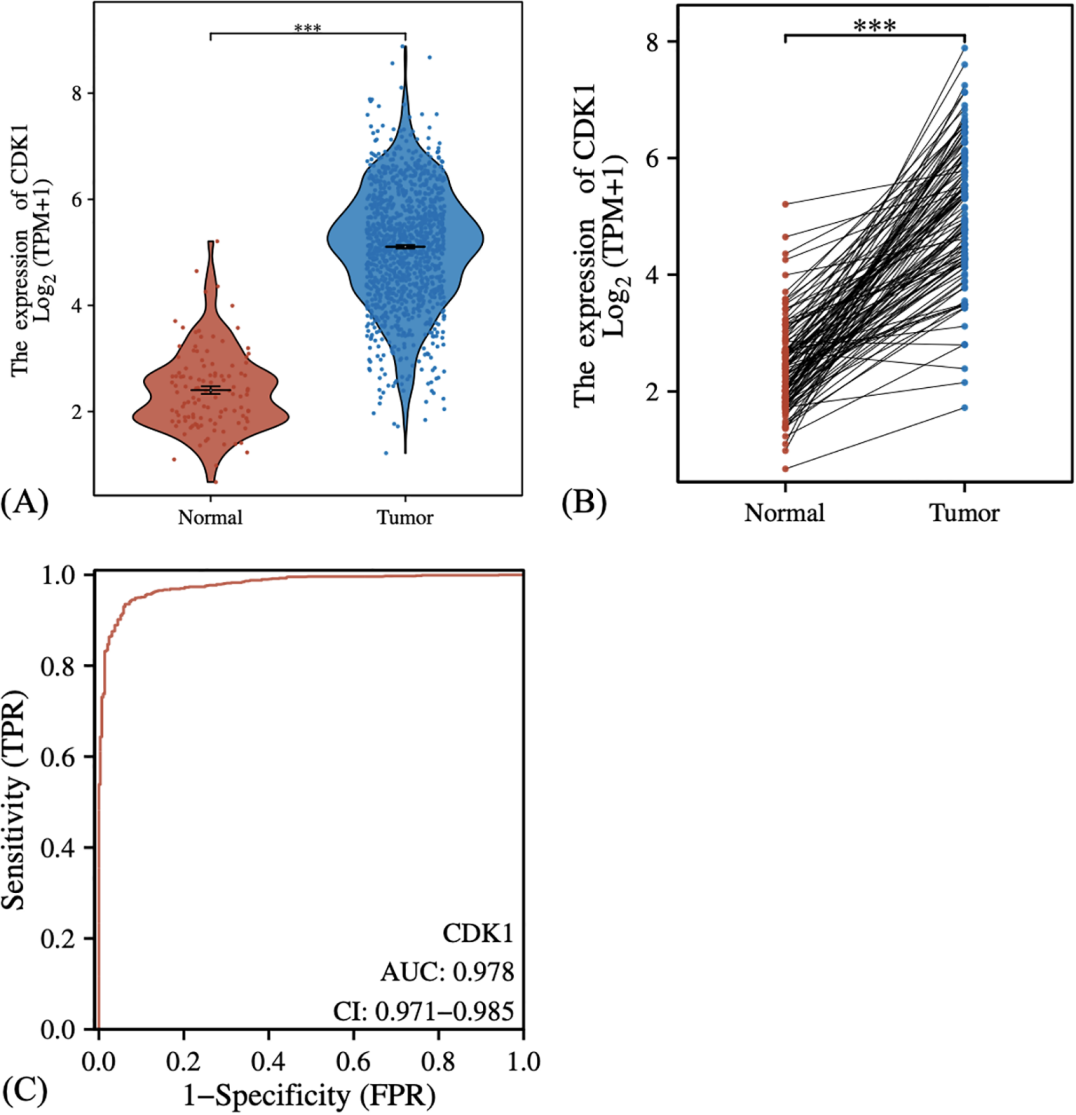
Expression analysis of CDK1 in normal and tumor tissues. **(A)** Comparison of CDK1 expression between normal and tumor tissues, showing higher expression in tumor tissues. **(B)** Paired analysis of CDK1 expression in normal versus tumor tissues, with most tumor samples exhibiting increased expression. **(C)** ROC curve demonstrating the high predictive accuracy of CDK1 for distinguishing between normal and tumor tissues. Statistical analysis was performed using the Wilcoxon rank-sum test (unpaired) and Wilcoxon signed-rank test (paired). ***: P-values <0.001.

First, we compared the expression levels of CDK1 in normal tissues and tumor tissues by analyzing the RNA-sequencing data (Level 3) for the breast cancer cohort from TCGA. In normal tissues, the expression of CDK1 is concentrated in a lower range, while the expression in tumor tissues is upregulated (**Figure 2A**). Paired analysis further demonstrated the expression differences between normal and tumor tissues in each pair of samples, with CDK1 expression generally higher in tumor samples (**Figure 2B**). We then used ROC curves to evaluate the predictive performance of CDK1 in tumor diagnosis and found that CDK1 showed a high classification ability with an AUC up to 0.978 (**Figure 2C**).

### CDK1 dysregulated in breast cancer cell line

**Figure 3:**
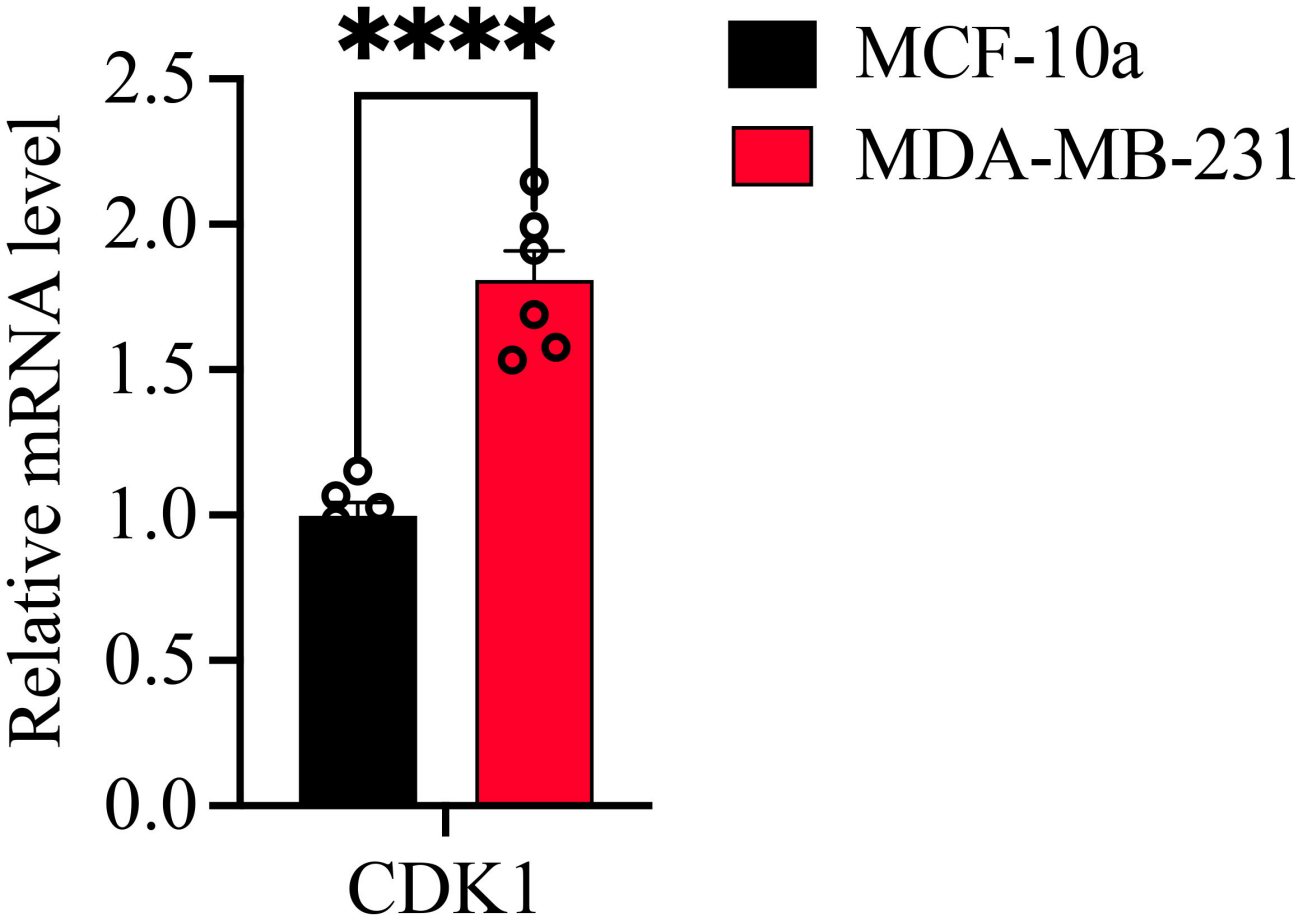
Expression of CDK1 in MCF-10a and MDA-MB-231 cell lines. Statistical significance was determined using student’s t-test. ****: P-values <0.0001.

We found that the high expression of CDK1 in the breast cancer cell line (MCF-7) was different from that in the normal breast epithelial cell line (MDA-MB-231), suggesting its potentially important role in breast cancer (**Figure 3**).

### CDK1 may play an important role in the molecular subtyping of breast cancer

**Figure 4:**
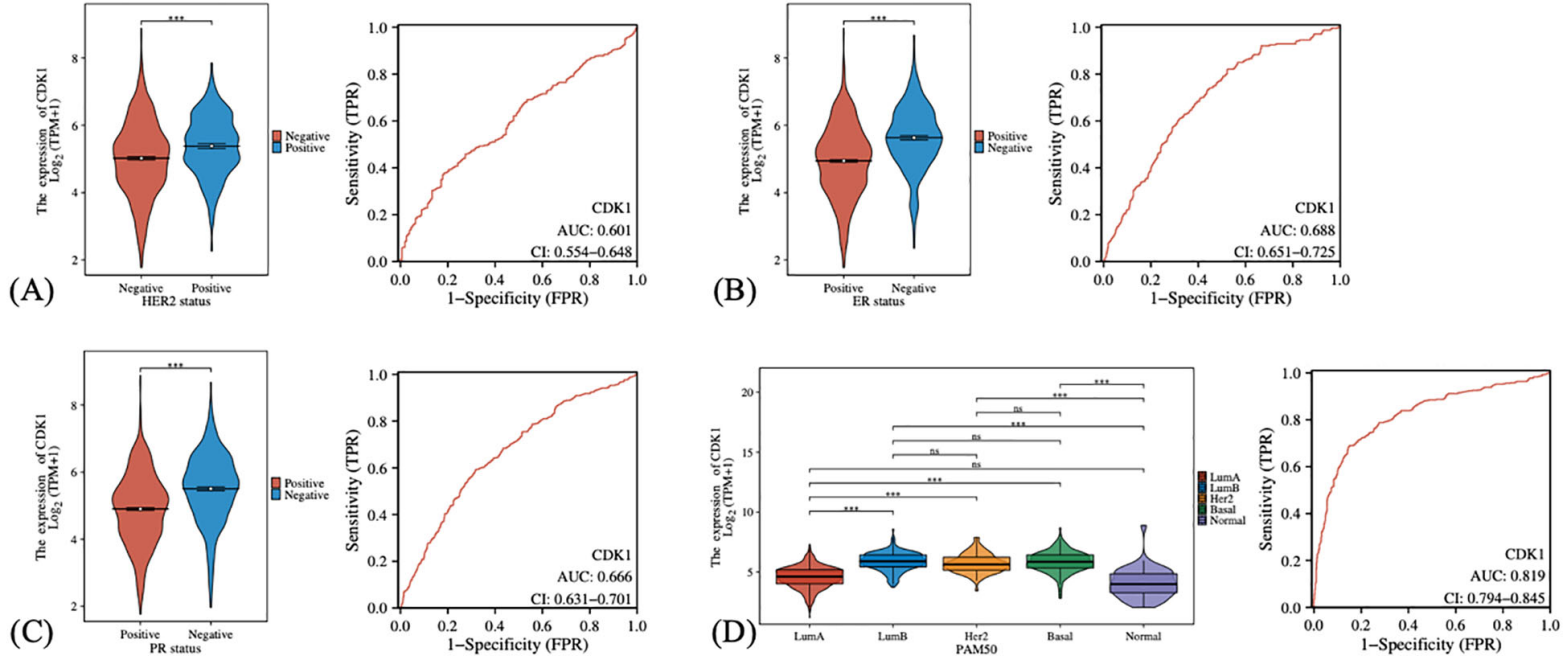
The expression differences and diagnostic efficacy of CDK1 in different clinical characteristics and subtypes of breast cancer. **(A)** Comparison of CDK1 expression in HER2-positive and HER2-negative patients. **(B)** CDK1 expression in estrogen receptor (ER)-positive and estrogen receptor (ER)-negative patients. **(C)** CDK1 expression in progesterone receptor (PR)-positive and progesterone receptor (PR)-negative patients. **(D)** CDK1 expression in different PAM50 subtypes. Statistical analysis was performed using the Wilcoxon rank-sum test (for two-group comparisons) or the Kruskal–Wallis test (for multi-group comparisons). ***: P-values <0.001. ns, Not significant.

We also investigated the expression levels of CDK1 in different breast cancer subgroups and its diagnostic efficacy. In the analysis of HER2 status (**Figure 4A**), it was found that the expression level of CDK1 in HER2-negative patients was higher than that in HER2-positive patients, suggesting that CDK1 may play a more important role in HER2-negative breast cancer. Similarly, the expression of CDK1 was elevated in the estrogen receptor (ER) and progesterone receptor (PR) negative groups (**Figures 4B, C**). ER-negative and PR-negative breast cancer usually have a poor prognosis, and high expression of CDK1 may be closely related to the biological characteristics of these malignant subtypes. The expression of CDK1 also showed obvious distribution differences in different PAM50 breast cancer subtypes (**Figure 4D**). The Basal subtype has the highest CDK1 expression levels, while the LumA subtype and normal-like tissues have the lowest expression. This differential expression pattern further suggests that CDK1 may play an important role in the molecular subtyping of breast cancer.

### Survival indicators in high and low CDK1 expression groups

**Figure 5:**
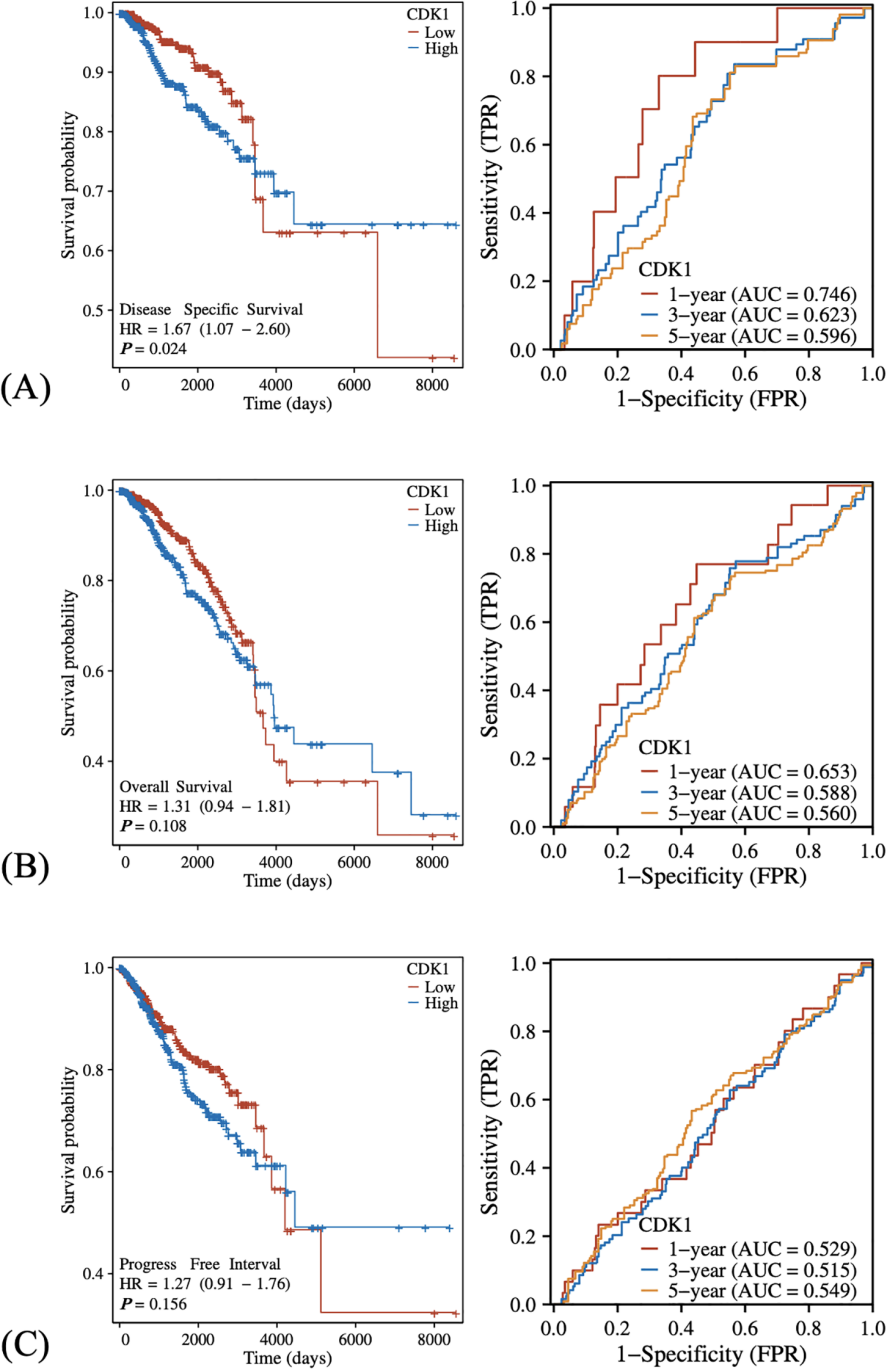
Differences in different survival indicators between patients with high and low expression of CDK1. **(A–C)** The left panels demonstrating the KM curves upon high and low CDK1 expression levels in DSS, OS, and PFI, respectively, while the right panels demonstrating the matching ROC analyses in 1, 2, and 3 years, respectively. Kaplan–Meier survival curves were compared using the log-rank test, and hazard ratios (HR) were calculated by univariate Cox regression.

The disease-specific survival (DSS) of patients with high CDK1 expression was worse than that of patients with low expression, with a hazard ratio (HR) of 1.67, and the difference was statistically significant (**Figure 5A**). The ROC curve further demonstrates the predictive ability of CDK1 at different time points. The 1-year AUC value is 0.746, and the 3-year and 5-year AUC values ​​are 0.623 and 0.596 respectively.

Meanwhile, although the high expression group also showed a worse trend in overall survival (OS), it did not reach statistical significance (**Figure 5B**).

Similarly, the progression-free survival (PFI) analysis showed that the survival time of the high expression group was also shorter, but the difference was not significant (**Figure 5C**).

### CDK1 expression positively correlates with TMB and T cell proliferation

In order to further understand the role of CDK1 in breast cancer, especially its role in the tumor immune microenvironment, we analyzed the relationship between different immune cell subtypes and CDK1 expression. The correlation heat map shows that high expression of CDK1 is positively correlated with infiltration of various immune cells, especially CD4+ memory T cells, CD8+ T cells, etc. (**Figure 1A**). These immune cell types are often closely associated with anti-tumor immune responses, suggesting that high expression of CDK1 may affect breast cancer progression by regulating the activity of these immune cells. We further analyzed the differences in immune cell enrichment between CDK1 high and low expression groups (**Figure 1B**). The results show that the enrichment degree of various immune cells in the high expression group is increased. These observations support that CDK1 may affect the immune microenvironment and patient prognosis of breast cancer by regulating immune cell infiltration. Tumor mutation burden (TMB) is a measure of a tumor’s genetic complexity by assessing the total number of mutations in its genome. Through correlation analysis, it can be observed that there is a certain positive correlation between the high expression of CDK1 and TMB (**Figure 1C**). This means that CDK1 may have more active expression in tumors with high mutation load, suggesting that it may be associated with increased tumor mutation levels. In addition, single-cell RNA sequencing analysis revealed the specific expression pattern of CDK1 in different immune cell subsets, especially in CD8+ T cells and M1 macrophages, where the expression of CDK1 was upregulated. This further supports the potential role of CDK1 in regulating anti-tumor immune responses (**Figure 1D**).

### CDK1 knockdown suppresses AKT activation and cyclin expression, leading to reduced breast cancer cell proliferation

**Figure 6:**
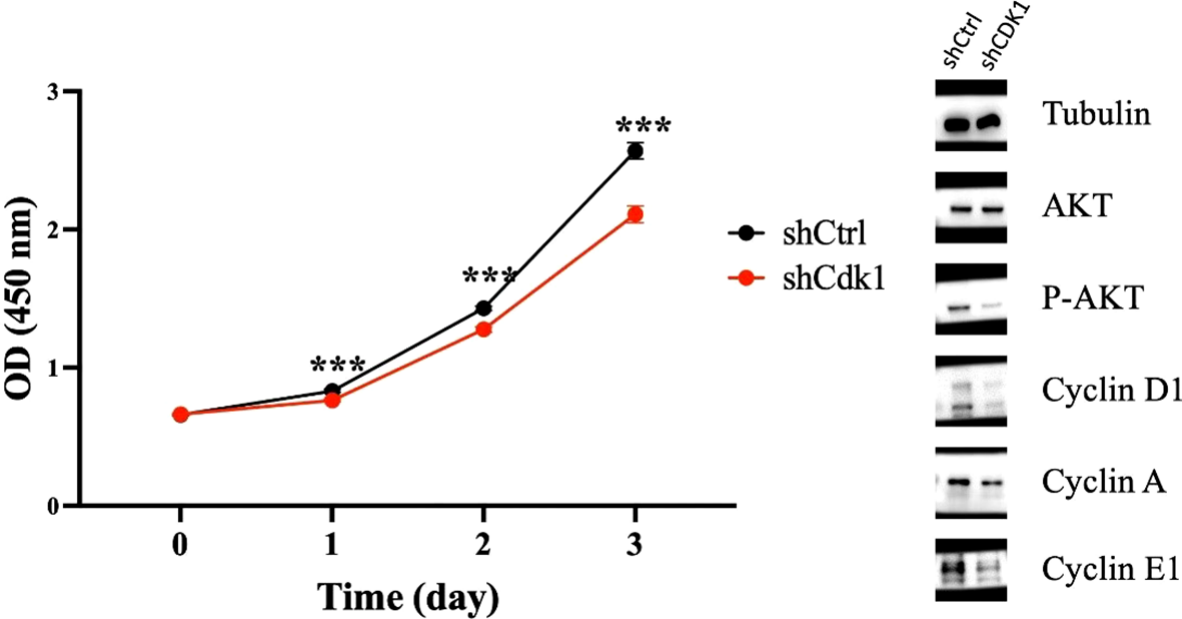
Knockdown of CDK1 decreases AKT phosphorylation and Cyclin D1/A/E1 expression, thereby inhibiting breast cancer cell proliferation. Statistical significance was determined using Student’s t-test; data are presented as mean ± SEM of three independent experiments. ***: P-values <0.001.

Knockdown of CDK1 did not alter the total AKT protein level but markedly reduced its phosphorylation. In parallel, Cyclin D1 expression was suppressed, followed by a decrease in Cyclin A and Cyclin E1 levels (**Figure 6**). Consistently, CCK-8 assays demonstrated that CDK1 knockdown impaired the proliferative capacity of breast cancer cells (**Figure 6**). Together, these findings indicate that CDK1 promotes breast cancer cell proliferation primarily by sustaining AKT activation and maintaining the expression of key cell cycle regulators. Original western blot images can be found in **Supplementary Material S1**.

## Discussion

In this study, we systematically analyzed the expression pattern, clinical relevance, and functional implications of CDK1 in breast cancer. Our data demonstrated that CDK1 is upregulated in tumor tissues compared with normal breast tissues, both in TCGA datasets and breast cancer cell lines. This consistent overexpression suggests that CDK1 may function as an oncogenic driver in breast cancer progression. The ROC analysis further confirmed its strong diagnostic potential, highlighting CDK1 as a promising biomarker for distinguishing malignant from normal tissues.

Notably, CDK1 expression was elevated in HER2-, ER-, and PR-negative subgroups, especially in the Basal-like subtype, which is typically characterized by poor prognosis and limited therapeutic options. This suggests that CDK1 may contribute to the aggressive biological behavior of these subtypes. Given the association between high CDK1 levels and worse disease-specific survival, CDK1 may serve as a valuable prognostic indicator. Although its impact on overall and progression-free survival did not reach statistical significance, the consistent trend toward poor outcomes in the high-expression group underscores its potential clinical importance. The lack of statistical significance for OS and PFI may reflect differences in endpoint definitions and follow-up duration, as DSS specifically measures tumor-related mortality, while OS and PFI can be influenced by non-cancer-related deaths and post-treatment variability.

Mechanistically, CDK1 appears to influence the tumor immune microenvironment (23–27). Our immune infiltration analysis revealed that CDK1 expression positively correlates with the abundance of multiple immune cell types (28–30). These findings imply that CDK1 might modulate immune cell activity, thereby shaping tumor-immune interactions. The observed positive correlation with TMB further indicates that CDK1 may be associated with genomic instability, a feature often linked to tumor aggressiveness and immunogenicity (31–36). Moreover, single-cell RNA-seq data revealed that CDK1 is preferentially expressed in proliferating T cells and M1 macrophages, reinforcing its possible role in immune regulation and tumor-host interactions. It is plausible that CDK1 may regulate immune cell recruitment through modulation of cell cycle–dependent cytokine expression or AKT-mediated immune signaling.

From a mechanistic standpoint, CDK1 knockdown reduced AKT phosphorylation and downregulated Cyclin D1, A, and E1, leading to suppressed breast cancer cell proliferation. This suggests that CDK1 promotes tumor growth primarily through sustaining AKT activation and maintaining the cell cycle machinery. The AKT pathway is a central regulator of cell proliferation and survival; thus, its suppression upon CDK1 knockdown provides a plausible explanation for the observed growth inhibition. These findings also align with prior studies reporting crosstalk between CDK1 activity and AKT signaling in various cancers.

Taken together, our results indicate that CDK1 acts as a multifaceted oncogene in breast cancer, contributing to tumor progression through both cell-intrinsic and immune-related mechanisms. The integration of transcriptomic, clinical, and functional evidence underscores CDK1’s potential as a diagnostic and therapeutic target. Nevertheless, further validation in larger clinical cohorts and mechanistic studies are needed to clarify the upstream regulatory factors and downstream effectors of CDK1. Due to funding and resource limitations, the current study focused mainly on *in vitro* assays. Future research involving *in vivo* xenograft models and rescue experiments will be essential to further substantiate the causal relationship between CDK1 and AKT signaling. In addition, exploring pharmacological inhibitors targeting CDK1 or its associated pathways could provide novel therapeutic strategies, particularly for patients with triple-negative or Basal-like breast cancer.

## Conclusion

In conclusion, CDK1 overexpression is closely associated with malignant progression, poor survival, immune infiltration, and AKT-driven proliferation in breast cancer. Targeting CDK1 may therefore represent a promising avenue for both prognostic assessment and therapeutic intervention in this disease.

## Data Availability

The datasets presented in this study can be found in online repositories. The names of the repository/repositories and accession number(s) can be found in the article/**Supplementary Material**.
