# Supplementary material for: CDK1 may promote breast cancer progression through AKT activation and immune modulation
Source: Front Oncol. 2025 Nov 6;15:1591706. doi: 10.3389/fonc.2025.1591706 (PMC12630994; doi:10.3389/fonc.2025.1591706)

**CDK1 May Promote Breast Cancer Progression through AKT Activation and Immune Modulation**

Huanhong Zeng ^a, #^, Minxue Zhuang ^a, #^, Bochuan Liang^b^, Feili Cai ^a^, Mengbo Lin ^a^, Ruijuan Wang^a, *^, Ruo Wang ^a, *^, Hui Zhang^a, *^

a Shengli Clinical Medical College of Fujian Medical University, Department of Breast Surgery, Fujian Provincial Hospital, Fuzhou University Affiliated Provincial Hospital, Fuzhou University, Fuzhou, China

b Nanchang Medical College, Nanchang, China

# These authors have contributed equally to this work

* Correspondence: Ruijuan Wang(wangruijuan@fzu.edu.cn); Ruo Wang(wangruo@fzu.edu.cn); Hui Zhang([drzhanghui@fjmu.edu.cn](mailto:drzhanghui@fjmu.edu.cn))

**Supplementary material S1_Original western blot images.**


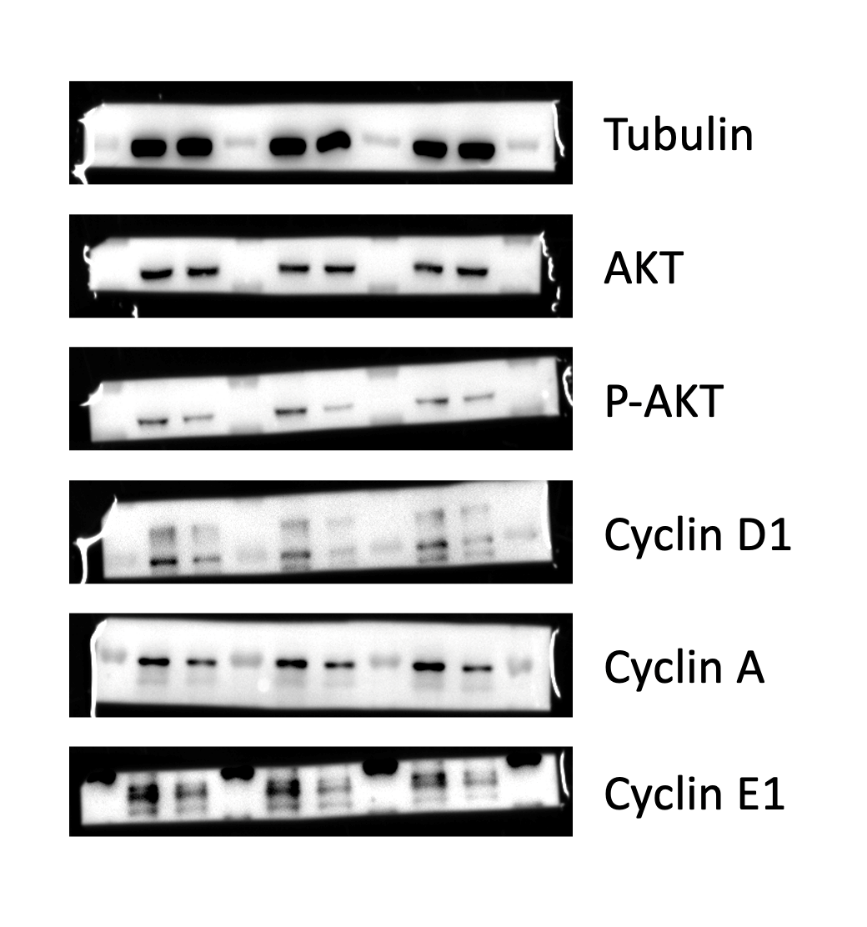

Supplement: Supplementary file 1 [file DataSheet1.docx]
